# Thyroid autoimmunity and IVF/ICSI outcomes in euthyroid women: a systematic review and meta-analysis

**DOI:** 10.1186/s12958-020-00671-3

**Published:** 2020-11-25

**Authors:** Ashley Venables, Wilbert Wong, Mandy Way, Hayden Anthony Homer

**Affiliations:** 1 Faculty of Medicine, The University of Queensland, Brisbane, Queensland Australia; 2 Faculty of Medicine, The University of Queensland, Brisbane, Queensland Australia; 3 QIMR Berghofer Medical Research Institute, Herston, Queensland Australia; 4 Christopher Chen Oocyte Biology Research Laboratory, UQ Centre for Clinical Research, The University of Queensland, Brisbane, Queensland Australia; 5 Reproductive Endocrinology and Infertility Clinic, Royal Brisbane & Women’s Hospital, Brisbane, Queensland Australia; 6 Queensland Fertility Group, Brisbane, Queensland Australia

**Keywords:** Thyroid autoimmunity, TAI, IVF, ICSI, ART, TSH, Pregnancy outcomes

## Abstract

**Background:**

Thyroid autoimmunity (TAI) – the presence of anti-thyroid peroxidase and/or anti-thyroglobulin antibodies – affects 8–14% of reproductively-aged women. It is hotly debated whether TAI adversely affects IVF/ICSI outcomes. This systematic review and meta-analysis evaluated the relationship between thyroid autoimmunity (TAI) and IVF/ICSI outcomes, both overall and amongst euthyroid women of known age using strict criteria for grouping pregnancy outcomes.

**Methods:**

The review was registered with PROSPERO: CRD42019120947. Searches were undertaken in MEDLINE, EMBASE, Web of Science and Cochrane Database from Inception-March 2020. Primary outcomes were clinical pregnancy rate, clinical miscarriage rate, biochemical pregnancy loss, livebirth rate per-cycle and live birth rate per clinical pregnancy (CP).

**Results:**

14 studies were included in the meta-analysis. Compared with women who tested negative for thyroid autoantibodies (TAI-), there was no significant difference in clinical pregnancy rate overall (OR 0.86; 95%CI [0.70, 1.05]; *P* = 0.14; 11 studies; I^2^ = 29.0%), or in euthyroid women (OR 0.88; 95%CI [0.69, 1.12]; *P* = 0.29; 10 studies; I^2^ = 32.0%). There was also no significant difference in clinical miscarriage rate overall (OR 1.04; 95%CI [0.52, 2.07]; *P* = 0.908; 8 studies; I^2^ = 53%), or in euthyroid women (OR 1.18; 95%CI [0.52, 2.64]; *P* = 0.69; 7 studies; I^2^ = 54%). There was no significant difference in biochemical pregnancy loss (OR 1.14; 95%CI [0.48, 2.72]; *P* = 0.769; 4 studies; I2 = 0.0%), live birth rate per cycle (OR 0.84; 95%CI [0.67, 1.06]; *P* = 0.145; I^2^ = 1.7%), live birth rate per clinical pregnancy (OR 0.67; 95%CI [0.28, 1.60]; *P* = 0.369; I^2^ = 69.2%), both overall and in euthyroid women as all studies included consisted of euthyroid women only. There was also no significant difference in number of embryos transferred, number of oocytes retrieved, mean maternal age or TSH levels overall or in euthyroid women.

**Conclusion:**

The findings of the present study suggest that thyroid autoimmunity has no effect on pregnancy outcomes in euthyroid women alone, or in euthyroid women and women with subclinical hypothyroidism.

**Supplementary Information:**

The online version contains supplementary material available at 10.1186/s12958-020-00671-3.

## Background

Thyroid autoimmunity (TAI) is defined as the presence of antithyroid antibodies – anti-thyroid peroxidase (TPO-Ab) and/or anti-thyroglobulin (TG-Ab) antibodies – and is the most common autoimmune condition in women of reproductive age, with a prevalence of 8–14% [1, 2]. Significantly, TAI has an even higher prevalence of up to 20% in infertile women [3]. Even when thyroid function remains normal, TAI has been associated with adverse pregnancy outcomes including spontaneous miscarriage and preterm birth [1, 4, 5]. Ovarian stimulation used during IVF/ICSI places additional demands on thyroid function [6, 7], which may potentially expose women with TAI (TAI+) to additional risks. Consequently, there has been intense interest in whether TAI compromises outcomes during IVF/ICSI treatment.

Multiple systematic reviews have investigated the relationship between TAI and IVF/ICSI outcomes, however there is substantial discrepancy between findings. Toulis et al. (2010) [8], found that sub-fertile TAI+ women undergoing IVF were at significantly greater risk of miscarriage than women without TAI (TAI-) but did not identify differences in either clinical pregnancy rates (CPR) or delivery rates. Thangaratinam et al. (2011) [9] also found a significantly higher miscarriage rate (MR) amongst TAI+ women. TAI+ women were found to have a greater risk of spontaneous miscarriage in addition to lower live birth rates (LBR) with no reductions in either CPR or implantation rates (IR) [6]. On the contrary, He et al. (2016) [10] found no association between TAI positivity and clinical pregnancy, miscarriage rate or delivery rate. Likewise, Leiva et al. (2017) [11] found no association between TAI and increased risk of pregnancy loss.

For a number of reasons, it is important that this topic be revisited. Firstly, earlier meta-analyses combined outcomes that were defined differently in different studies. For instance, some papers considered miscarriage to include both clinical and biochemical pregnancy losses (BPL) (e.g. Muller et al. 1999 [12], Poppe et al. 2003 [13]) whereas others included only clinical losses (e.g. Chai et al. 2014 [14]; Lucaszuk et al. 2015 [15]). In the most recent comprehensive meta-analysis on the effect of TAI on IVF outcomes, all miscarriage types, whether biochemical or clinical were pooled into a single analysis [6]. For uniformity, it is critically important to analyse each miscarriage type separately since biochemical and clinical losses have very different frequencies and aetiologies [16]. Biochemical pregnancy loss in IVF is estimated to have an incidence of up to 22% [17], compared to clinical miscarriage which has an incidence of ~ 15% [18, 19]. A number of mechanisms have been proposed by which, TAI might increase miscarriage risk including detrimental effects on the endometrium, embryo toxicity, general immune disturbance and compromised ability of thyroid function to satisfy increased demands of pregnancy [2]. It is conceivable that very early post-implantation pregnancy stages might have a different vulnerability to these threats than later stages. To explore whether stage-specific vulnerability to TAI might exist, we not only analysed outcomes for all miscarriages, but also differentiated between biochemical and clinical losses whenever possible. For LBR analyses, some papers calculated LBR on a per-cycle basis whereas others calculated on a per-clinical pregnancy (CP) basis; again, these were combined into a single analysis [6]. As with MR outcomes, it is important that like-for-like livebirth outcomes are compared. Notably, similar concerns also apply to the two meta-analyses on this topic performed prior to 2016 [8, 9].

A second critically important consideration is that female age was not reported in 3 of the 12 studies included in the most recent comprehensive meta-analysis [6]. This is pivotal since arguably the most important determinant of pregnancy outcome is female age due to the effects of ageing on oocyte quality, which ultimately impacts aneuploidy rates [20]. Moreover, when age was analysed for the 9 papers in which maternal age was known, Busnelli et al. (2016) [6] found that TAI+ women were significantly older than TAI- women. Based on these analyses, therefore, it is very unclear to what extent the older age of the TAI+ cohort contributed to the adverse reproductive outcomes that were observed.

Thirdly, since the last meta-analysis, at least 4 further studies analysing women of known ages have been published [21–24]. Busnelli et al. (2016) [6] included a total of 428 TAI+ women of known ages. These 3 newer studies [21–24] contain an additional 390 TAI+ women.

Fourthly, half of the 12 studies included in Busnelli et al. (2016) [6] were published prior to 2010. In more recent years, guidelines from the ASRM and the American Thyroid Association [25] suggest that a lower thyroid stimulating hormone (TSH) cut-off (2.5 mIU/mL) should be used for initiating levothyroxine treatment in TAI+ women. Newer studies may therefore include women with more intact thyroid function whereas older studies may have included patients with more severe thyroid imbalances, which may have increased the tendency to adverse outcomes in earlier meta-analyses. Indeed, two of the studies in the recent meta-analysis [12, 26] included women with overtly abnormal TSH levels. It is notable in this regard that the 2011 meta-analysis identified an increased odds ratio (OR) for miscarriage of 3.15 amongst TAI+ women [9] whereas in Busnelli et al. (2016) [6], the OR had declined to less than half this at 1.44 [6]. Furthermore, in He et al. (2016) [10] and Leiva et al. (2017) [11], no significant relationship was found between TAI and IVF outcomes. While He et al. (2016) [10] found no association between TAI positivity and clinical pregnancy, miscarriage rate or delivery rate in euthyroid women, they did find that women with subclinical hypothyroidism (SCH) and TAI were at greater risk of miscarriage and had a lower delivery rate than TAI- women, indicating that the extent of associated thyroid dysfunction, rather than TAI per se, could account in some part for adverse outcomes reported in previous studies.

Here we undertook an updated systematic review and meta-analysis of the overall relationship between TAI and IVF/ICSI outcomes as has been done previously, as well as separate analyses of euthyroid women of known female age using strict criteria for defining pregnancy outcomes.

## Materials and methods

### Protocol and registration

PRISMA guidelines were followed and the review was registered with PROSPERO (Registration no. CRD42019120947).

### Search strategy

Literature searches were conducted via MEDLINE, EMBASE, The Cochrane Library (Cochrane Database of Systematic Reviews, Cochrane Central Register of Controlled Trials (CENTRAL), Cochrane Methodology Register), and Web of Science (Science and Social Science Citation Index) from inception to April 2019. Searches were rerun in March 2020 prior to publication. The Medline search utilised a combination of text and medical subject headings (MeSH): (“Thyroid Gland”[Mesh] AND “Autoimmunity”[Mesh]) OR “thyroid autoimmunity” OR “thyroid autoantibody” OR “thyroid autoantibodies” OR antithyroid peroxidase OR “antithyroglobulin antibody”, “Fertilization in Vitro”[Mesh] OR IVF OR “invitro fertilization” OR “in vitro fertilization” OR “in-vitro fertilization” OR “invitro fertilization” OR “invitro fertilisation” OR “in vitro fertilisation” OR “in-vitro fertilisation” OR “invitro fertilisation” OR “Sperm Injections, Intracytoplasmic”[Mesh] OR “intracytoplasmic sperm injection” OR ICSI OR ART [tiab] OR “reproductive techniques, assisted”[MeSH Terms] OR “assisted reproductive technology”. Search terms were set by the authors and were adapted for use with other databases. No language restrictions were implemented. Peer reviewed case control and cohort studies were eligible for inclusion.

### Study selection

Title and abstract screening were performed independently by two authors (A.V. and W.W.) to eliminate studies deemed irrelevant. The full text versions of relevant studies were retrieved and assessed for eligibility by the same author. Any uncertainty was resolved through discussion with a third author. Studies were excluded if: (i) women had overt thyroid dysfunction, (ii) maternal age was not reported, (iii) women were being treated with levothyroxine, (iv) ART techniques aside from IVF/ICSI were used. The quality of the studies was assessed using the Newcastle Ottawa scale [27].

### Data extraction and analysis

Two authors extracted data independently. Study year, setting, design, thyroid antibody assays used and cut-off values, number of TAI+ and TAI- patients, number of IVF/ ICSI cycles performed, type of ART procedure performed and key outcomes of the study were recorded. Extracted data was compared to ensure accuracy, and discrepancies were corrected upon referral back to the original paper.

The primary outcomes measured were CPR, MR, and LBR as; (i) CP: the presence of a gestational sac on ultrasound scan, CPR: the proportion of IVF/ICSI cycles resulting in a CP; (ii) MR: the proportion of pregnancies that miscarried/did not result in a delivery or live birth, CMR: the proportion of clinical pregnancies that miscarried/did not result in a delivery or live birth, BPL: the proportion of biochemical pregnancies that were lost prior to the CP stage (iii) LBR: the proportion of cycles of IVF/ICSI that resulted in the birth of at least one live baby, calculated per cycle and per CP. In cases where outcomes of interest were not reported by the study but could be determined from the raw data, outcomes were calculated manually. Secondary outcomes such as overall MR (including all definitions of MR: BPR + CMR + CMR alone), maternal age, number of oocytes retrieved (NOR), and numbers of embryos transferred (ET) were also recorded.

### Statistical method

Data were analysed in STATA 15 [28]. Pooled effect sizes were estimated by applying random effects meta-analysis. The I^2^ statistic was used as a measure of heterogeneity between studies. An I^2^ value of 0% indicates no heterogeneity whereas values of 25, 50 and 75% reflect low, moderate and high heterogeneity, respectively [29]. Egger’s weighted regression test was used to assess publication bias where more than 10 studies were included in the analysis. Sensitivity analyses were performed where moderate levels of heterogeneity were detected.

## Results

### Results of literature search and description of included studies

**Fig. 1:**
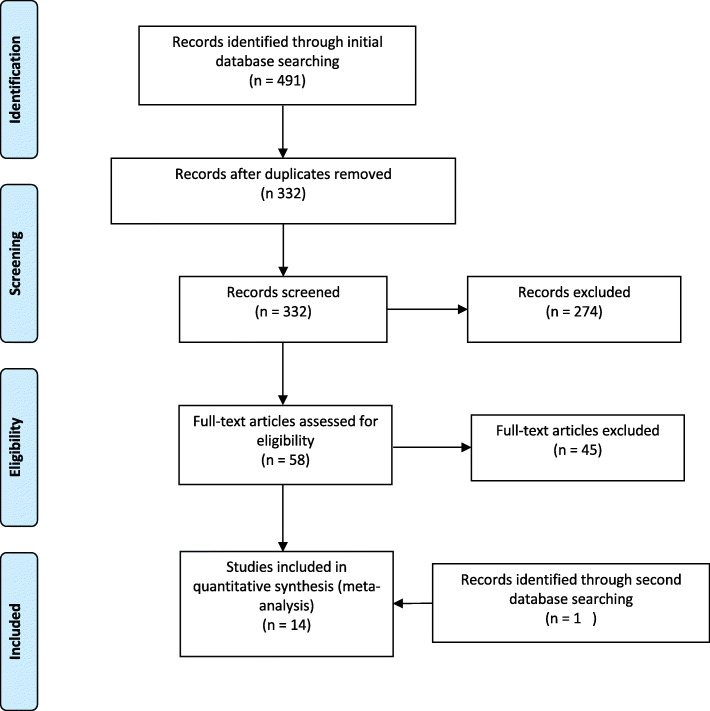
Flowchart depicting study selection process

Systematic searches of the PubMed, Embase, Web of Science and Cochrane Library databases identified 491 citations, with 332 of these being non-duplicates (Fig. 1). 274 articles were excluded based on title and abstract screening. The full texts of 58 articles were retrieved. An additional 45 articles were excluded (Fig. 1) on the following basis: 5 articles did not report maternal age, 7 articles did not include either a TAI+ group or a TAI- control group, 3 studies included patients on levothyroxine, 3 were review articles, 3 did not report patient outcomes, 19 were conference abstracts, 1 did not use IVF/ICSI, 2 were excluded due to patient overlap and 2 studies were excluded during data analysis due to discrepancies in results (additional file 1). One more study was added in March 2020 when searches were rerun. Details of the 14 studies that were included in the final analyses are presented in Table 1.

**Table 1:** Description of included studies investigating the association between TAI status and IVF/ICSI outcomes

Study	Year	Study Population	Study Design	Thyroid autoantibodies tested and cut-off values	Assay used for thyroid antibodies	TAI+ patients	TAI+ TSH Levels	TAI- patients	TAI- TSH Values	ART procedure	Outcomes	Quality of evidence ^a^
Muller [12]	1999	Women undergoing IVF March 1994–March 1996	Prospective	TPO-Ab: normal range < = 80 IU/ml	RIA	25	3.5+/−3.6	148	1.7+/−1.7	IVF	MR	7
Poppe [13]	2003	Women undergoing ART October 1999–November 2000	Prospective	TPO-Ab: normal range < 100 KU/L	RIA	32	1.6 (0.02–4.1)	202	1.3 (0.05–3.6)	IVF/ICSI	CPR, CMR, BPL, MR, LBR/CP, ET	8
Negro [30]	2007	Women undergoing ART Jan 2000- Jan 2006	Retrospective Cohort	TPO-Ab: normal range < 100 KU/I	RIA	374	-	42	-	IVF/ICSI	MR, ET, NOR	7
Kilic [31]	2008	Women with unexplained fertility undergoing first ART procedure Jan 2006-Feb 2007	Prospective Cohort	TPOAb: normal< 34 IU/ml, Tg Ab: normal < 150 IU/ml	Electrochemiluminescence	23	2.21+/−1.25	31	1.99+/− 1.11	IVF	CPR, BPL, NOR	7
Revelli [32]	2009	Women undergoing ART Feb 2004 – May 2008	Retrospective Cohort	TPO-Ab: normal range < 35 U/ml TG-Ab: normal range < 40 IU/ml	Immunofluorescence	38 (52 cycles)	2+/− 1.2	200	2.2+/− 1.1	IVF	CPR, CMR, MR, NOR	8
Zhong [33]	2012	Women undergoing ART Aug 2009 - Aug 2010	Retrospective Cohort	TPO-Ab: normal range < 5.61 IU/ml TG-Ab: normal range < 4.11 IU/ml	CMIA	90 (156 cycles)	–	676 (981 cycles)	–	IVF/ICSI	MR, IR, ET, NOR	8
Karacan [34]	2013	Infertile women undergoing ICSI May 2009–July 2012	Prospective cohort	TPO-ab: normal < 35 IU/ml Tg-Ab: normal< 115 IU/ml	ECLIA	34	1.89+/− 1.18	219	1.74+/− 1.24	ICSI	CPR, CMR, BPL, MR, IR, ET, NOR	8
Chai [14]	2014	Infertile women undergoing first IVF/ICSI cycle between Feb 2007 and Dec 2009*	Retrospective cohort	TPO-ab: normal < 9 IU/ml Tg-Ab: normal< 4 IU/ml	TPO antibody reagent pack	89	1.5 (0.35–2.47)	419	1.4 (0.32–2.50)		CPR, CMR, MR, LBR/CP	7
Tan [35]	2014	Women undergoing ICSI 1997–2006	Retrospective	TPO-ab: normal < 35 U/L Tg-Ab: normal < 40 U/L	Chemiluminescence	110	1.7+/− 0.7	725	1.5+/− 0.6	ICSI	CPR, CMR, MR, LBR/CP	8
Lukaszuk [15]	2015	Women undergoing ART April 2010–April 2012	Retrospective	TPO-ab: normal < 34 IU/mL	ECLIA	114	All euthyroid	495	All euthyroid	ICSI	CPR, CMR, MR, LBR/CP, IR	7
Sakar [23]	2016	Women undergoing ICSI July 2013–March 2014	Prospective case-control	TPO-ab: normal < 35 U/L Tg-Ab: normal < 78 U/L	ELISA	31 (32 cycles)	2.35+/− 1.84	121 (126 cycles)	1.97+/− 2.07	ICSI	CPR, CMR, BPL, MR, NOR	8
Chen [21]	2017	Infertile women Jan 2015-Dec 2015**	prospective cohort	TPO-Ab: normal range < 34kIU/L	ECLIA	214	-	844	-	IVF/ ICSI	CPR, CMR, IR, ET, NOR	8
Zhuang [24]	2017	Women undergoing ICSI Mar 2011-Oct 2013***	Retrospective cohort	TPO-Ab, TG-Ab Cut-off values not reported	Not Reported	127	All euthyroid	662	All euthyroid	IVF	CPR, NOR	7
Devi [22]	2019	Women undergoing IVF Nov 2014- Apr 2016	Prospective cohort	TPO-ab: normal < 50 IU/ml Tg-Ab: normal < 100 IU/ml	ELISA	17	All euthyroid	64	All euthyroid	IVF	CPR, NOR	8

^a^Assessed using the Newcastle Ottawa Scale

*Only euthyroid women included

**Only women with TPO-ab were included to prevent sample overlap

***Only women with TSH ≤ 2.5mIU/L included

CPR, clinical pregnancy rate; CMR, clinical miscarriage rate; BPL, biochemical pregnancy loss; MR; miscarriage rate according to all definitions; LBR/CP, live birth rate per clinical pregnancy; LBR/ cycle, live birth rate per cycle; IR, implantation rate; ET, numbers of embryos transferred; NOR, number of oocytes retrieved

### Outcomes

#### CPR

**Fig. 2:**
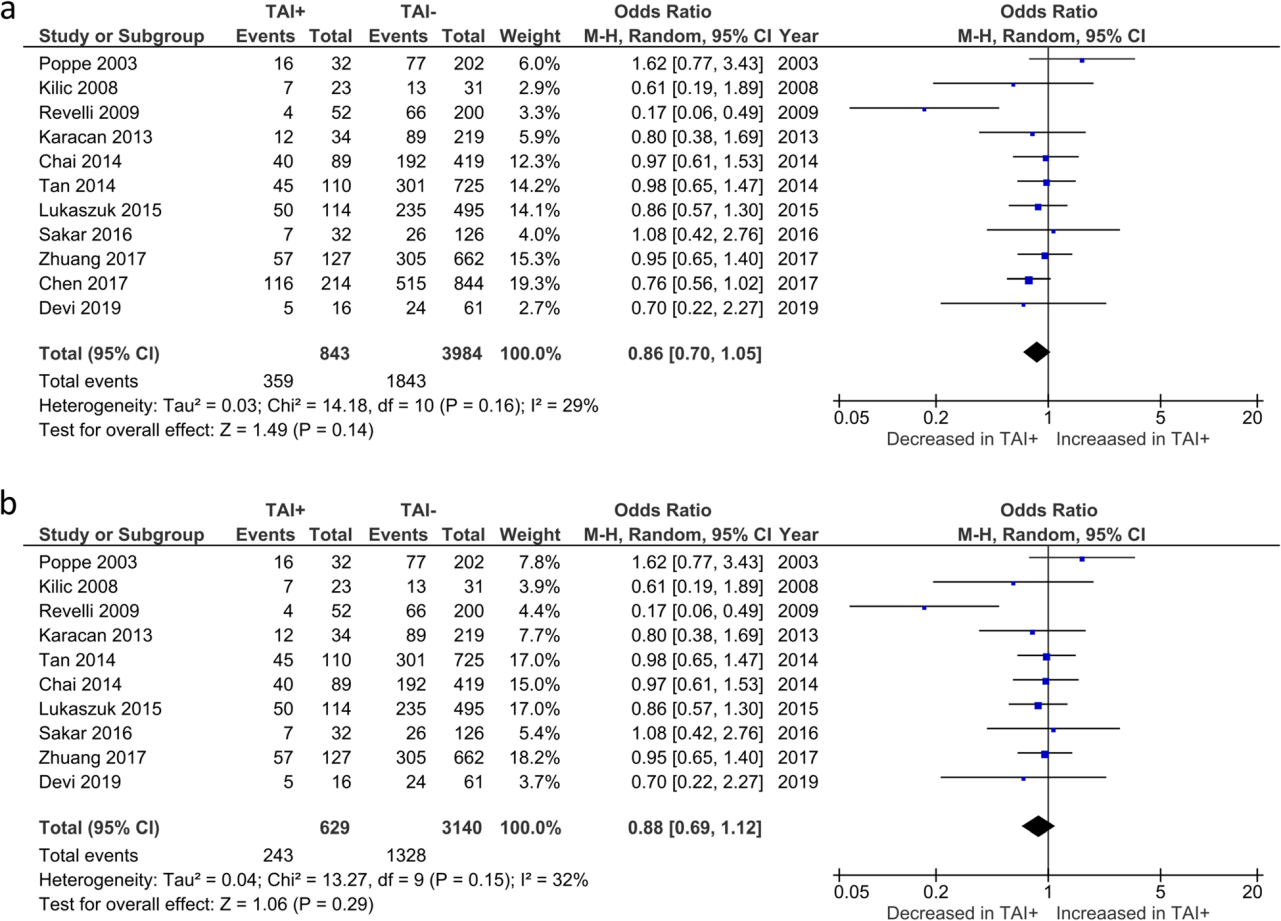
**a** Overall association between thyroid autoimmunity and likelihood of clinical pregnancy. Results showed no significant difference in clinical pregnancy rates between TAI+ and TAI- women overall (OR 0.86; 95%CI [0.70, 1.05]; P = 0.14; 11 studies; I^2^ = 29.0%). **b** Association between thyroid autoimmunity and likelihood of clinical pregnancy in euthyroid women. Results showed no significant difference in clinical pregnancy rates in TAI+ and TAI- euthyroid women (OR 0.88; 95%CI [0.69, 1.12]; P = 0.29; 10 studies; I^2^ = 32.0%). TAI+, thyroid autoimmune; TAI-, not thyroid autoimmune; OR, odds ratio; CI, confidence interval

Overall CPR was reported by 11 studies. No significant difference in CPR was observed between TAI+ and TAI- women (OR 0.86; 95%CI [0.70, 1.05]; *P* = 0.14; 11 studies; I^2^ = 29.0%)(Fig. 2a). CPR in euthyroid women was reported by 10 studies. No significant difference in CPR was observed between TAI+ and TAI- women (OR 0.88; 95%CI [0.69, 1.12]; *P* = 0.29; 10 studies; I^2^ = 32.0%)(Fig. 2b).

#### MR

**Fig. 3:**
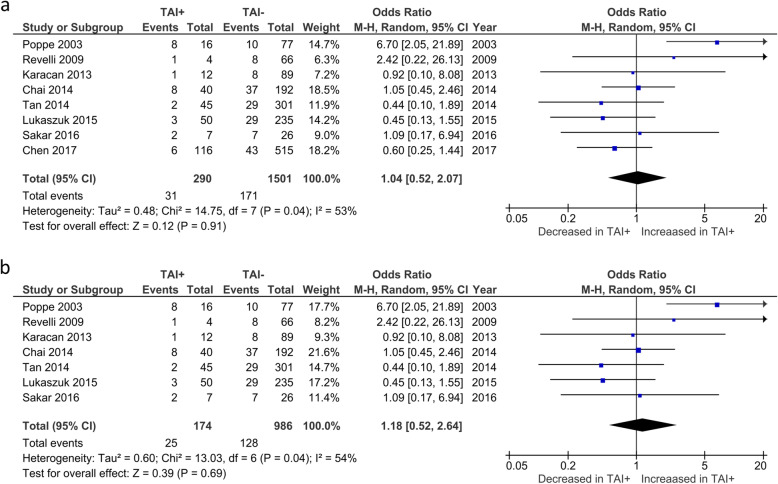
**a** Overall association between thyroid autoimmunity and clinical miscarriage rate. Results showed no significant difference in clinical miscarriage rate between TAI+ and TAI- women overall (OR 1.04; 95%CI [0.52, 2.07]; P = 0.908; 8 studies; I^2^ = 53%). b) Association between thyroid autoimmunity and clinical miscarriage rate in euthyroid women. Results showed no significant difference in clinical miscarriage rate between TAI+ and TAI- euthyroid women (OR 1.18; 95%CI [0.52, 2.64]; P = 0.69; 7 studies; I^2^ = 54%). TAI+, thyroid autoimmune; TAI-, not thyroid autoimmune; OR, odds ratio; CI, confidence interval

**Fig. 4:**
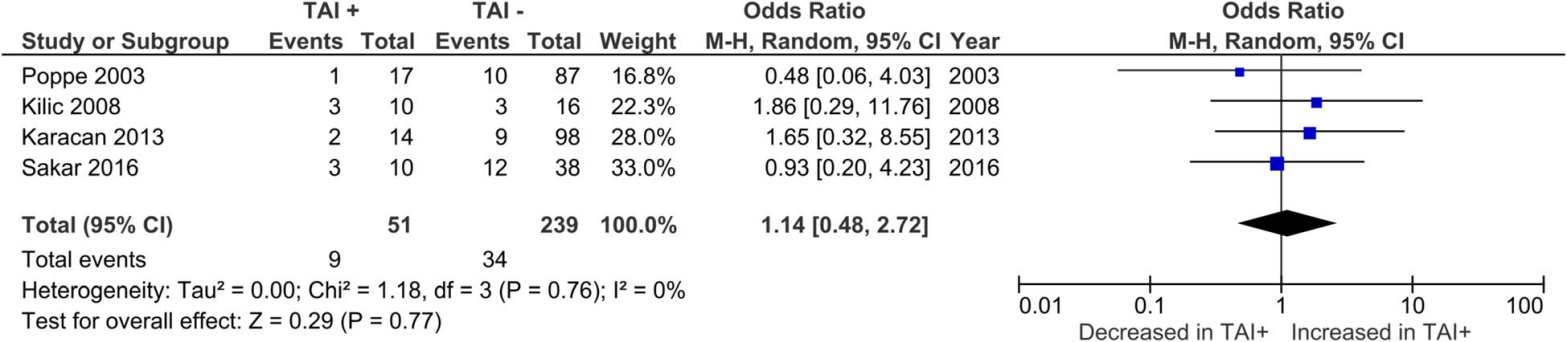
Association between thyroid autoimmunity and likelihood of biochemical pregnancy loss. Results showed no significant difference in biochemical pregnancy loss between TAI+ and TAI- women overall (OR 1.14; 95%CI [0.48, 2.72]; *P* = 0.77; 4 studies; I^2^ = 0.0%). TAI+, thyroid autoimmune; TAI-, not thyroid autoimmune; OR, odds ratio; CI, confidence interval

**Fig. 5:**
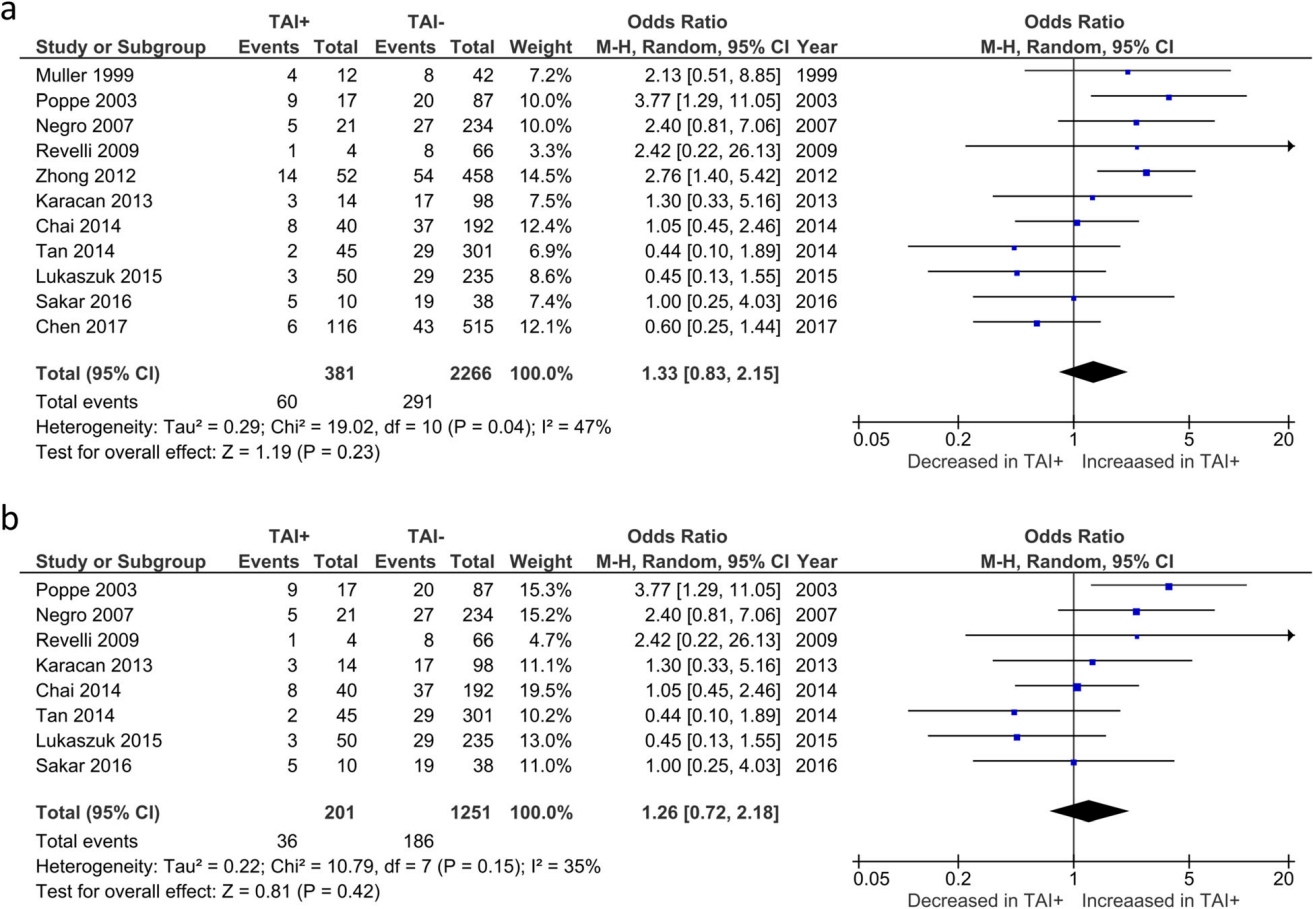
Overall association between thyroid autoimmunity and likelihood of miscarriage according to all definitions. Results showed no significant difference in likelihood of miscarriage according to all definitions between TAI+ and TAI- women overall (OR 1.33; 95%CI [0.83–2.15]; P = 0.23; 11 studies; I^2^ = 47.4%). b) Association between thyroid autoimmunity and likelihood of miscarriage according to all definitions in euthyroid women. Results showed no significant difference in likelihood of miscarriage according to all definitions between TAI+ and TAI- euthyroid women (OR 1.26; 95%CI [0.72–2.18]; P = 0.42; 8 studies; I^2^ = 35%). TAI+, thyroid autoimmune; TAI-, not thyroid autoimmune; OR, odds ratio; CI, confidence interval

Overall CMR was reported by 8 studies. No significant difference was observed in CMR between TAI+ and TAI- women (OR 1.04; 95%CI [0.52, 2.07]; *P* = 0.908; 8 studies; I^2^ = 53%) (Fig. 3a). CMR in euthyroid women was reported by 7 studies. No significant difference was observed in CMR between TAI+ and TAI- women (OR 1.18; 95%CI [0.52, 2.64]; *P* = 0.69; 7 studies; I^2^ = 54%) (Fig. 3b). BPL was reported by 4 studies, all of which only included euthyroid women (Fig. 4). No significant difference was observed in BPL between TAI+ and TAI- women (OR 1.14; 95%CI [0.48, 2.72]; *P* = 0.769; 4 studies; I2 = 0.0%). 11 studies reported overall MR using a variety of definitions. No significant difference in MR based on all definitions combined was observed (OR 1.33; 95%CI [0.83–2.15]; *P* = 0.23; 11 studies; I^2^ = 47%)(Fig. 5a). 8 studies reported MR using a variety of definitions in euthyroid women. No significant difference in MR based on all definitions combined was observed (OR 1.26; 95%CI [0.72–2.18]; *P* = 0.42; 8 studies; I^2^ = 35%)(Fig. 5b).

#### LBR

**Fig. 6:**
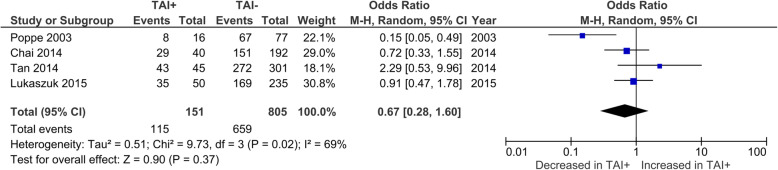
Association between thyroid autoimmunity and likelihood of live birth per clinical pregnancy. Results showed no significant difference in likelihood of live birth per clinical pregnancy between TAI+ and TAI- women overall (OR 0.67; 95%CI [0.28, 1.60]; *P* = 0.37; 4 studies; I^2^ = 69%). TAI+, thyroid autoimmune; TAI-, not thyroid autoimmune; OR, odds ratio; CI, confidence interval

**Fig. 7:**
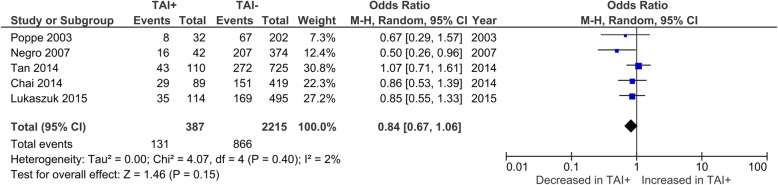
Association between thyroid autoimmunity and likelihood of live birth per cycle. Results showed no significant difference in likelihood of live birth per cycle between TAI+ and TAI- women overall (OR 0.84; 95%CI [0.67, 1.06]; *P* = 0.15; 5 studies; I^2^ = 2%)**.** TAI+, thyroid autoimmune; TAI-, not thyroid autoimmune; OR, odds ratio; CI, confidence interval

LBR per CP was reported by 4 studies, all of which only included euthyroid women. No significant difference was observed in LBR between TAI+ and TAI- women (OR 0.67; 95%CI [0.28, 1.60]; *P* = 0.369; I^2^ = 69.2%)(Fig. 6). LBR per cycle was reported by 5 studies and no significant difference was observed in LBR per cycle between TAI+ and TAI- women (OR 0.84; 95%CI [0.67, 1.06]; *P* = 0.145; I^2^ = 1.7%) (Fig. 7).

#### ET

**Fig. 8:**
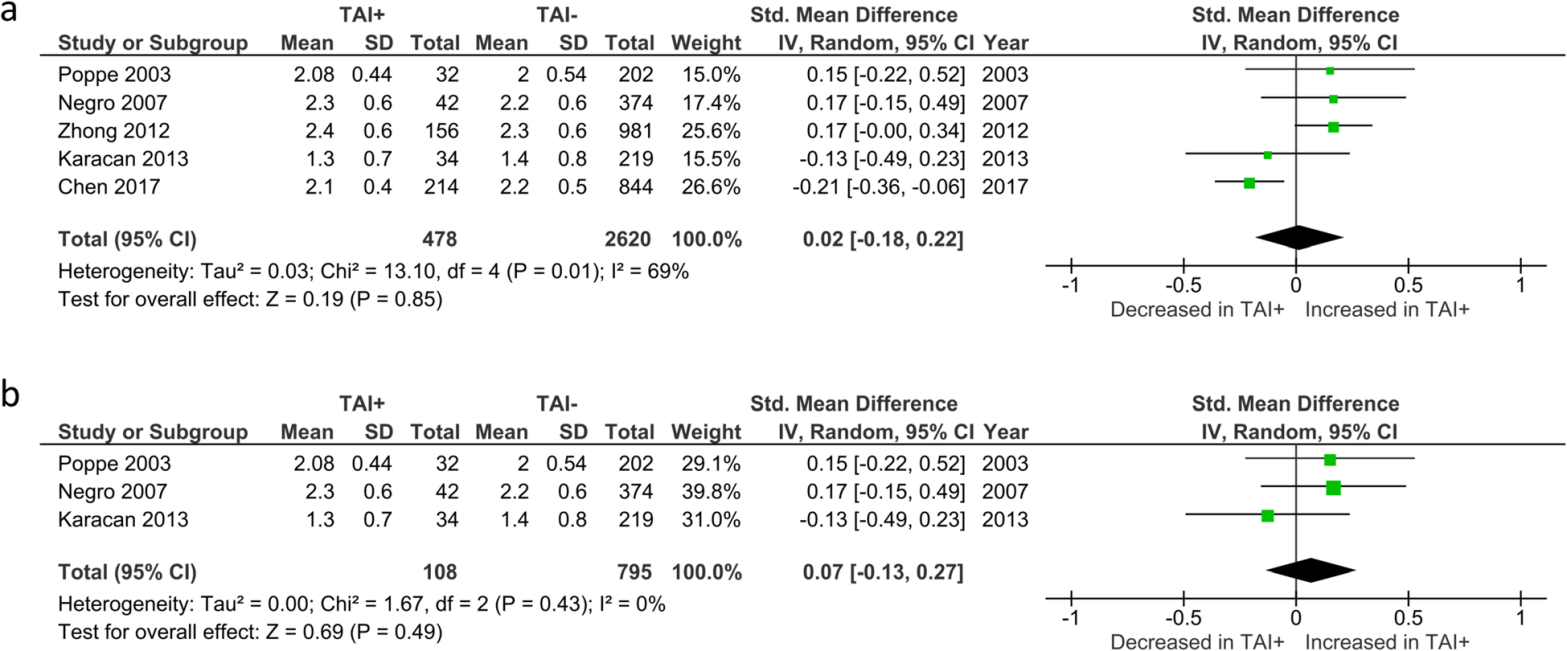
**a** Overall association between thyroid autoimmunity and mean number of embryos transferred. Results showed no significant difference in number of embryos transferred between TAI+ and TAI- women overall (SMD 0.02; 95%CI [− 0.18, 0.22]; *P* = 0.85; 5 studies; I^2^ = 69%). **b** Association between thyroid autoimmunity and mean number of embryos transferred in euthyroid women. Results showed no significant difference in number of embryos transferred between TAI+ and TAI- euthyroid women (SMD 0.07; 95%CI [− 0.13, 0.23]; *P* = 0.49; 3 studies; I^2^ = 0%). TAI+, thyroid autoimmune; TAI-, not thyroid autoimmune; SMD, standard mean difference; CI, confidence interval

Overall mean number of embryos transferred was reported by 5 studies. No significant difference in mean number of transferred embryos was observed between TAI+ and TAI- women (SMD 0.02; 95%CI [− 0.18, 0.22]; *P* = 0.849; I^2^ = 69%) (Fig. 8a). Mean number of embryos transferred in euthyroid women was reported by 3 studies. No significant difference in mean numbers was observed between TAI+ and TAI- women (SMD 0.07; 95%CI [− 0.13, 0.27]; *P* = 0.49; I^2^ = 0%) (Fig. 8b).

#### NOR

**Fig. 9:**
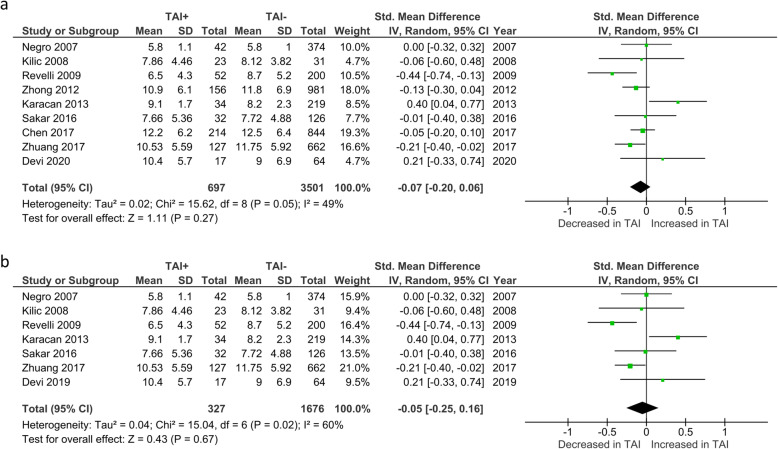
**a** Overall association between thyroid autoimmunity and number of oocytes retrieved. Results showed no significant difference in number of oocytes retrieved between TAI+ and TAI- women overall (SMD -0.07; 95%CI [− 0.20, 0.06]; *P* = 0.27; 9 studies; I^2^ = 49%). **b** Association between thyroid autoimmunity and number of oocytes retrieved in euthyroid women. Results showed no significant difference in number of oocytes retrieved between TAI+ and TAI- euthyroid women (SMD -0.05; 95%CI [− 0.25, 0.16]; *P* = 0.67; 7 studies; I^2^ = 60%). TAI+, thyroid autoimmune; TAI-, not thyroid autoimmune; SMD, standard mean difference; CI, confidence interval

Overall number of oocytes retrieved (NOR) was reported by 9 studies. No significant difference was observed between TAI+ and TAI- women (SMD -0.07; 95%CI [− 0.20, 0.06]; *P* = 0.27; 9 studies; I^2^ = 49%)(Fig. 9a). NOR in euthyroid women was reported by 7 studies. No significant difference was observed between TAI+ and TAI- women (SMD -0.05; 95%CI [− 0.25, 0.16]; *P* = 0.67; 7 studies; I^2^ = 60%)(Fig. 9b).

#### Age

**Fig. 10:**
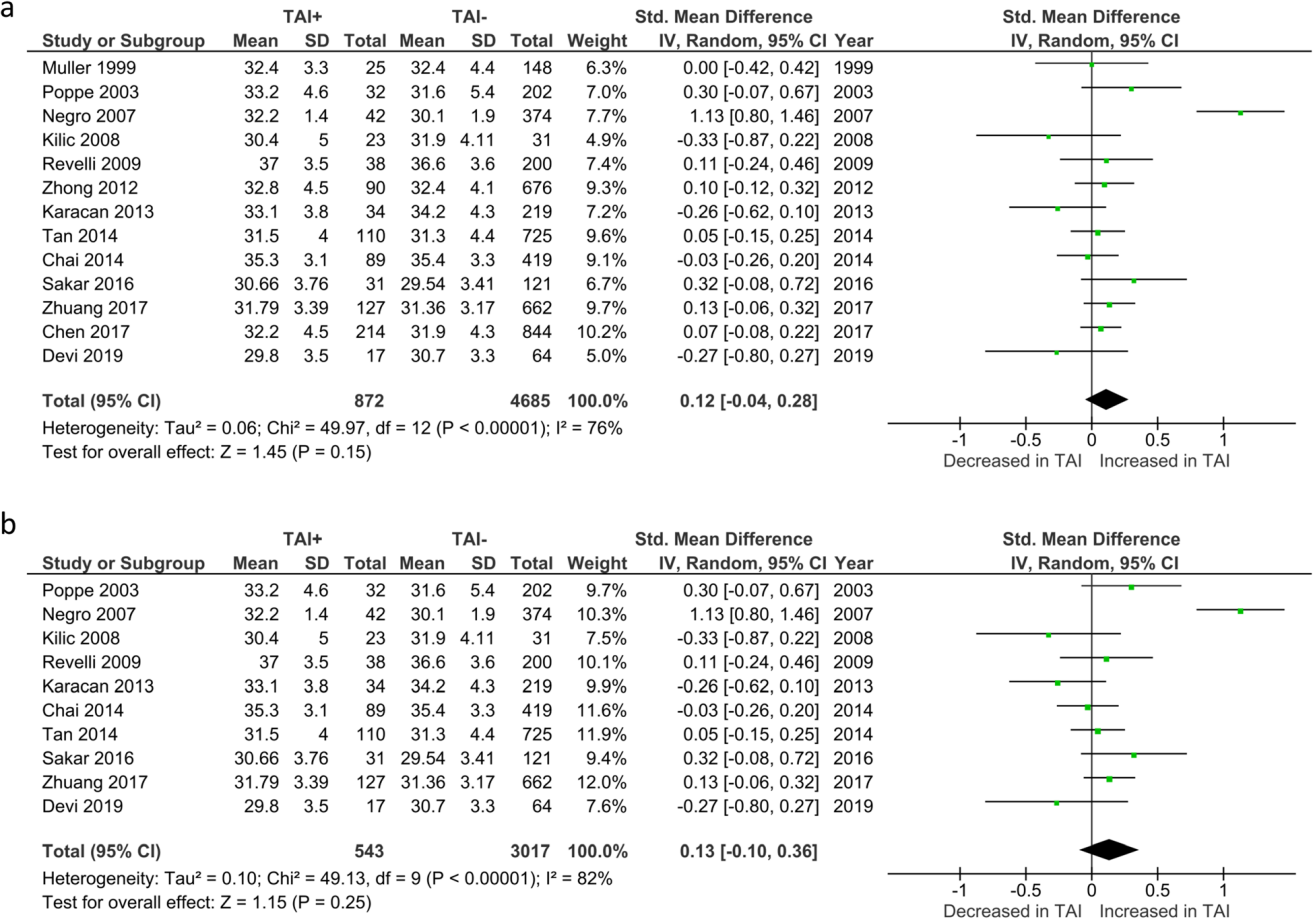
**a** Overall association between thyroid autoimmunity and mean maternal age. Results showed no significant difference in mean maternal age between TAI+ and TAI- women overall (SMD 0.12; 95%CI [− 0.04, 0.28]; P = 0.15; 13 studies; I^2^ = 76%). **b** Association between thyroid autoimmunity and mean maternal age in euthyroid women. Results showed no significant difference in mean maternal age between TAI+ and TAI- euthyroid women (SMD 0.13; 95%CI [− 0.10, 0.36]; *P* = 0.25; 10 studies; I^2^ = 82%). TAI+, thyroid autoimmune; TAI-, not thyroid autoimmune; SMD, standard mean difference; CI, confidence interval

Age was reported by all 14 studies. One study reported age as the median (25th–75th) [15] and could not therefore be included in our analysis of mean ages. However, since patients in this study had a median age below 37 without any significant difference between TAI+ and TAI- groups [15], data from this study was included in other analyses. Analysis of the 13 remaining studies revealed no significant differences in patient age between all TAI+ and TAI- women (SMD 0.12; 95%CI [− 0.04, 0.28]; *P* = 0.15; 13 studies; I^2^ = 76%) (Fig. 10a). There were also no significant age differences between the sub-group of TAI+ and TAI- women who were euthyroid (SMD 0.13; 95%CI [− 0.10, 0.36]; *P* = 0.25; 10 studies; I^2^ = 82%)(Fig. 10b).

#### TSH

**Fig. 11:**
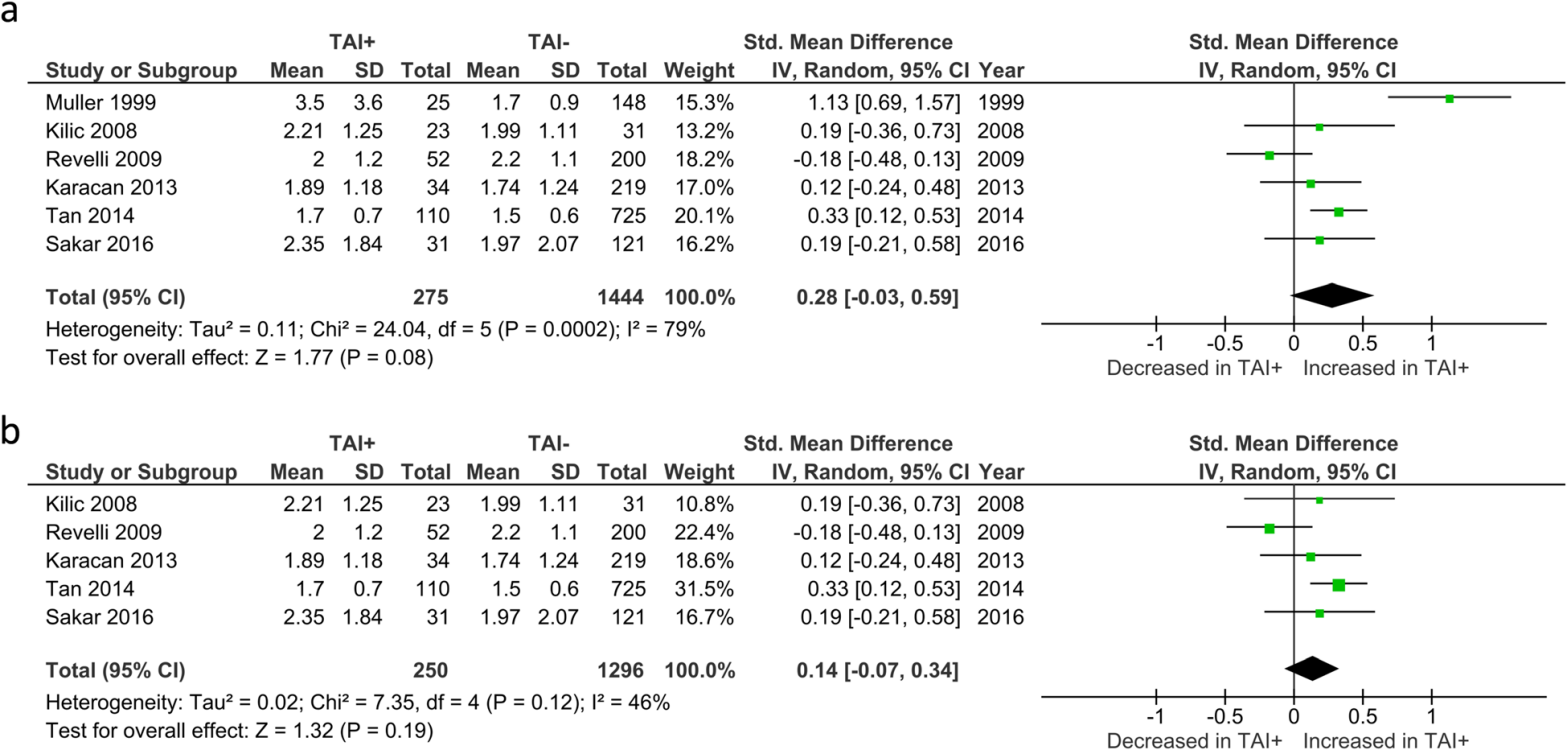
**a** Overall association between thyroid autoimmunity and TSH levels. Results showed no significant difference in TSH levels between TAI+ and TAI- women overall (SMD 0.28; 95%CI [− 0.03, 0.59]; *P* = 0.08; 6 studies; I^2^ = 79%). **b** Association between thyroid autoimmunity and TSH levels in euthyroid women. Results showed no significant difference in TSH levels between TAI+ and TAI- euthyroid women (SMD 0.14; 95%CI [− 0.07, 0.34]; *P* = 0.19; 5 studies; I^2^ = 46%). TAI+, thyroid autoimmune; TAI-, not thyroid autoimmune; SMD, standard mean difference; CI, confidence interval

TSH levels were reported by 6 studies and no significant difference was seen between TAI+ and TAI- women (SMD 0.28; 95%CI [− 0.03, 0.59]; *P* = 0.08; 6 studies; I^2^ = 79%)(Fig. 11a). In the 5 studies that reported TSH levels specifically in euthyroid women, no significant difference in TSH levels was seen between TAI+ and TAI- women (SMD 0.14; 95%CI [− 0.07, 0.34]; *P* = 0.19; 5 studies; I^2^ = 46%)(Fig. 11b).

#### Heterogeneity and sensitivity analysis

Analyses displaying moderate/high heterogeneity (I^2^ ≥ 50%) were adjusted following sensitivity analysis (Table 2). Removal of 1–2 studies was sufficient to reduce heterogeneity in all cases. Following sensitivity analyses, CPR, CMR, MR, LBR per CP and ET showed no change in statistical significance outcomes compared with unadjusted analyses. In NOR analyses, exclusion of Karacan et al. (2013) [34] led to significantly fewer oocytes being retrieved amongst TAI+ women (SMD -0.13; 95%CI [− 0.22, − 0.04], *P* = 0.007).

**Table 2:** Results of meta-analysis before and after sensitivity analysis

*Outcome*	Unadjusted OR / SMD (95% CI)	*P* value	I^2^	Studies Excluded Following Sensitivity Analysis	OR (95% CI) Following Exclusion of Studies due to Sensitivity Analysis	*P* value	I^2^
***CPR - overall***	OR 0.86; 95%CI [0.70, 1.05]	0.14	29%				
***CPR - euthyroid***	OR 0.88; 95%CI [0.69, 1.12];	0.29	32%				
***CMR – overall***	OR 1.04; 95%CI [0.52, 2.07]	0.908	53%	Poppe 2003	OR 0.74; 95%CI [0.46, 1.19)	0.21	0.00%
***CMR – euthyroid***	OR 1.18; 95%CI [0.52, 2.64]	0.69	54%	Poppe 2003	OR 0.81; 95%CI [0.46, 1.41]	0.45	0.00%
***BPL- overall***	OR 1.14; 95%CI [0.48, 2.72]	0.769	0%				
***BPL- euthyroid***	-						
***MR - overall***	OR 1.33; 95%CI [0.83, 2.15]	0.23	47%				
***MR - euthyroid***	OR 1.26; 95%CI [0.72–2.18]	0.42	35%				
***LBR/CP - overall***	OR 0.67; 95%CI [0.28, 1.60]	0.37	69%	Poppe 2003	OR 0.92; 95%CI [0.57, 1.48]	0.722	0.00%
***LBR/CP - euthyroid***	–						
***LBR/cycle - overall***	OR 0.84; 95%CI [0.67, 1.06]	0.15	2%				
***LBR/cycle – euthyroid***	-						
***ET - overall***	SMD 0.02; 95%CI [−0.18, 0.22]	0.85	69%	Chen 2017	SMD 0.13; 95%CI [−0.00, 0.26]	0.054	0.00%
***ET - euthyroid***	SMD 0.07; 95%CI [− 0.13, 0.2]	0.49	0%				
***NOR – overall***	SMD −0.07; 95%CI [− 0.20, 0.06]	0.27	49%				
***NOR - euthyroid***	SMD −0.05; 95%CI [− 0.25, 0.16]	0.67	60%	Karacan 2013	SMD −0.14; 95%CI [− 0.30, 0.02]	0.08	26%
***Age - overall***	SMD 0.12; 95%CI [−0.04, 0.28]	0.15	76%				
***Age - euthyroid***	SMD 0.13; 95%CI [−0.10, 0.36]	0.25	82%				
***TSH - overall***	SMD 0.28; 95%CI [−0.03, 0.59]	0.08	79%				
***TSH – euthyroid***	SMD 0.14; 95%CI [−0.07, 0.34]	0.19	46%				

CPR, clinical pregnancy rate; CMR, clinical miscarriage rate; BPL, biochemical pregnancy loss; MR; miscarriage rate according to all definitions; LBR/CP, live birth rate per clinical pregnancy; LBR/ cycle, live birth rate per cycle; IR, implantation rate; ET, mean number of embryos transferred; NOR, number of oocytes retrieved

#### Publication Bias

Results of Egger’s weighted regression test indicated no publication bias in mean maternal age, CPR, overall MR or OPR.

#### Quality analysis

Of the 14 studies included, the quality ranged from 7 to 8 (Table 1). No studies were excluded from the analysis based on poor quality.

## Discussion

Here we undertake the most comprehensive meta-analysis to-date regarding the effect of TAI on IVF/ICSI outcomes in women of known ages and TSH status. For the first time, we use strict criteria to analyse BPL and CMR as well as LBR on a per-cycle and per-CP basis in euthyroid women as well as in a larger group, which included women with SCH. We found no difference in any pregnancy outcomes in relation to TAI status. Our results contrast with previous meta-analyses showing increased MR and/or decreased LBR in TAI+ women overall and in euthyroid women undergoing IVF/ICSI [6, 8, 9]. On the other hand, our findings are consistent with those of He et al. (2016) [10] and Leiva et al. (2017) [11]. There are important differences between our study and previous meta-analyses that may explain some of these discrepancies. Our systematic review included the most recent studies such as Devi et al. (2019) [22], Chen et al. (2017) [21] and Sakar et al. (2016) [18], which did not find any association between TAI positivity and pregnancy outcome. We also analysed outcomes in studies that were strictly limited to euthyroid women in addition to the wider group seen in previous studies. We were also strict in analysing like-for-like outcomes (e.g. CMR and LBR per cycle) rather than combining variously defined outcomes into a single analysis. Given the powerful influence of female age on pregnancy outcome through effects on oocyte quality [20], a critically important strength of our study was to restrict analyses to papers that reported female age. Importantly, we found that both groups were comparably aged thereby unequivocally ruling out the possibility of an age effect. In contrast, in the most recent meta-analysis, one quarter of included studies had not reported age and analysis of ages amongst those that did, found that TAI+ women were significantly older than TAI- women [6]. A meta-regression analysis performed by the authors suggested that such differences were unlikely to have impacted their outcomes [6]. However, this cannot be concluded with certainty since, as the authors themselves noted, only 9 studies were included in the meta-regression, which is below the minimum of 10 recommended for avoiding Type II errors [29] added to which, ages were completely unknown for a substantial proportion of included studies. Furthermore, by considering maternal TSH levels, the present study accounted for potential confounding effects due to substantial thyroid dysfunction.

The lack of a detrimental effect of TAI on pregnancy outcomes that we find here is consistent with two recent large RCTs. The first RCT, the POSTAL study, tested whether levothyroxine supplementation could reduce MR in 600 TAI+ euthyroid women (mean age 31.6 years) undergoing IVF and found there was no benefit [36]. Since this study did not include a TAI- group, it is not known whether MR was actually increased in their untreated TAI+ cohort. Indeed, the study was powered against a predicted CMR of 30% for TAI+ women but unexpectedly, the actual MR in their untreated TAI+ arm was only 10.6%, which was no higher, and indeed, somewhat lower than the background rate of ~ 15% for women of a similar age [18, 19]. The recently reported TABLET trial [37] studied a broader population of TAI+ women with a history of miscarriage or infertility and again found no benefit of levothyroxine supplementation. Significantly, however, the rate of combined CMR and BPL was ~ 30%; this rate, which includes biochemical losses that occur with high frequency, also seems to be no higher than the predicted rate in the general population [16]. It is therefore likely that the lack of effect observed in both studies was at least in part because there isn’t a markedly increased risk of miscarriage in the first place in TAI+ women, entirely consistent with our present findings.

There has been another recent meta-analysis that did not consider the full breadth of IVF patients as we did here, but instead focused only on studies in which ICSI was performed for fertilisation [38]. Consequently, it only contained 4 studies, 3 of which had been included in the earlier meta-analysis by Busnelli et al. (2016) [6], along with a more recent paper [23] which we included in our study. Significantly, Poppe et al. (2018) [38] found no difference in MR when analyses were strictly restricted to CPs, entirely in keeping with our findings. There have been a number of alternative theories regarding ICSI’s ability to overcome the adverse effects of TAI on pregnancy outcomes. It has been suggested that anti thyroid antibodies may bind antigens in the zona pellucida, thereby disrupting its function [39], and that ICSI may be able to overcome this effect. However, in light of the present study’s findings, it appears more likely that the finding of Poppe et al. (2018) [38] are due to the inclusion of more recent studies and stricter inclusion criteria.

A recent meta-analysis investigating the effect of levothyroxine treatment found that MR in TAI+ women who conceived with ART were not reduced by levothyroxine but that levothyroxine decreased MR among TAI+ women who also had SCH [40] suggesting that SCH rather than TAI per se may confer the detrimental effect. Considering the recent change in guidelines, which now propose that TAI+ women with TSH > 2.5 mIU/mL should be supplemented with thyroxine [25, 41], most women with SCH and TAI were more likely to have been excluded from more recent studies. It is therefore possible that previous negative effects on pregnancy outcomes in TAI+ women may have been due to SCH, rather than TAI itself. It is worth noting that a number of studies included in previous analyses that were not included in the present study did not report serum TSH levels, making it possible that the relationship between TAI and adverse pregnancy outcomes may have been due to thyroid dysfunction. We note, however, that the results of the present study do not support this notion since we found no association between TAI and pregnancy outcome either in euthyroid women or the overall group. Studies such as Chai et al. (2014) [14] and Zhuang et al. (2017) [24] separated their investigation on TAI and IVF based on maternal serum TSH levels < 2.5 and > 2.5 and found no difference in IVF outcomes based on TSH status. Furthermore, A recent retrospective study and meta-analysis found no increased risk of adverse pregnancy outcomes in women with TSH > 2.5 [42]. Thus, whilst this idea may hold some merit, further investigation is required.

We acknowledge that a variety of factors may influence the strength of our findings. Individual studies assessed different antibodies (TPO-Ab and/or TG-Ab) and used different assays as well as different cut-off values for defining TAI positivity (Table 1), which could potentially affect outcomes. Cause of infertility maybe be a potential confounding factor. A number of the included studies reported no significant difference in cause of infertility between the TAI+ and TAI- groups. However, each had varying inclusion criteria, and in recent a recent meta-analysis, TAI has been shown to be more prevalent in conditions associated with poor IVF outcomes such as PCOS [43]. It has been shown that the presence of TPO-Ab in particular is associated with an increased risk of PCOS and endometriosis, and varying aetiologies based on the inclusion criteria and antibodies present may have influenced results [36]. There were also limited details on individual patients such as stimulation protocols used, which could theoretically affect results given that antagonist and agonist protocols could have different effects on thyroid function [44]. The numbers of embryos transferred could also impact pregnancy outcomes. Although we considered this potential confounder, and analysis of the five studies that reported embryo numbers transferred showed no difference in relation to TAI status (Fig. 8), we did not have data on embryo numbers for 9 studies. We note as well that although we adhered to uniform criteria for outcomes, the included studies defined and reported clinical outcomes differently and some studies were excluded based on discrepancies in the reported data, a lack of clarity regarding definitions, or insufficient available data regarding clinical outcomes. It therefore remains possible that some important data were omitted that may have influenced the results.

## Conclusions

The results of the present study suggest that TAI has no effect on pregnancy outcomes in women undergoing IVF either overall, or in euthyroid women. The findings of this study may be useful when deciding on treatment plans for TAI+ women undergoing IVF.

## Supplementary Information

**Additional file 1**

## Data Availability

The current study was based on results of relevant published studies.

## Abbreviations

TAI: Thyroid Autoimmunity
TPO-Ab: Anti-thyroid peroxidase antibodies
TG-Ab: Anti-thyroglobulin antibodies
MR: Miscarriage rate
CMR: Clinical miscarriage rate
BPL: Biochemical pregnancy loss
CP: Clinical pregnancy
CPR: Clinical pregnancy rate
LBR: Live birth rate
IR: Implantation rate
NOR: Number of oocytes retrieved
ET: Embryos transferred
TSH: Thyroid stimulating hormone
SCH: Subclinical hypothyroidism
OR: Odds ratio
CI: Confidence interval
