# Supplementary material for: Thyroid autoimmunity and IVF/ICSI outcomes in euthyroid women: a systematic review and meta-analysis
Source: Reprod Biol Endocrinol. 2020 Nov 25;18:120. doi: 10.1186/s12958-020-00671-3 (PMC7687721; doi:10.1186/s12958-020-00671-3)
Supplement: Supplementary file 1 — Additional file 1 [file 12958_2020_671_MOESM1_ESM.docx]

**Appendix 1: reasons for exclusion of studies**

| No age | Multiple a=Antibodies | Levothyroxine use | Review article | Outcomes not reported | Patient Overlap | Conference Abstract | Not TAI | Not IVF | Discrepancies in Data | |
| --- | --- | --- | --- | --- | --- | --- | --- | --- | --- | --- |
| Singh 1995 | Geva 1996 | Kim 1998 | Kutteh 2002 | Al-Mousley 2003 | Poppe 2004 | Reh 2010 | Kim 2011 | Seungdamrong 2017 | Litwicka 2014 | |
| Kutteh 1999 |  | Unuane 2016 | Poppe 2003 | Poppe 2003 | Negro 2007 | Akdogan 2012 | Ying 2012 |  | Adrisani 2018 | |
| Monteleone 2011 | | Medenica 2018 | Poppe 2004 | Bals-Pratsch 2005 | | Chi 2012 | Fumarola 2013 | |  |  |
| Mintziori 2014 | |  |  |  |  | Karacan 2012 | Weghofer 2015 | |  |  |
| Mintziori 2017 | |  |  |  |  | Kotori 2013 | Wang 2017 |  |  |  |
|  |  |  |  |  |  | Weghofer 2013 | Beydilli 2018 | |  |  |
|  |  |  |  |  |  | Mintziori 2014 | |  |  |  |
|  |  |  |  |  |  | Dokuzeylul 2015 | |  |  |  |
|  |  |  |  |  |  | Seungdamrong 2015 | |  |  |  |
|  |  |  |  |  |  | Vega 2015 |  |  |  |  |
|  |  |  |  |  |  | De Brucker 2016 | |  |  |  |
|  |  |  |  |  |  | Kim 2016 |  |  |  |  |
|  |  |  |  |  |  | Tabata 2016 | |  |  |  |
|  |  |  |  |  |  | Cao 2017 |  |  |  |  |
|  |  |  |  |  |  | Cao 2017 |  |  |  |  |
|  |  |  |  |  |  | Grove-Laugesen 2017 | |  |  |  |
|  |  |  |  |  |  | Araujo 2018 |  |  |  |  |
|  |  |  |  |  |  | Heshiki 2018 | |  |  |  |
|  |  |  |  |  |  | Chai 2013 |  |  |  |  |
|  |  |  |  |  |  |  |  |  |  |  |

References – appendix 1

1. Akdogan A, Demirtas O, Sahin G, Tavmergen E, Goker ENT. Does thyroid autoimmunity effect IVF success rates via follicular fluid. Human Reproduction. 2012;27.

2. Al-Mousley NA, Karaki RZ. Association of autoantibodies with repeated in-vitro fertilization and embryo transfer failure. Middle East Fertility Society Journal. 2003;8(1):60-4.

3. Andrisani A, Sabbadin C, Marin L, Ragazzi E, Dessole F, Armanini D, et al. The influence of thyroid autoimmunity on embryo quality in women undergoing assisted reproductive technology. Gynecol Endocrinol. 2018;34(9):752-5.

4. Araújo Filho E, De Araújo LP, De Marchi BR, Fácio CL, Machado-Paula LA, Previato LF. Incidence of acquired thrombophilia in Assisted Reproduction. Jornal Brasileiro de Reproducao Assistida. 2018;22(3):274.

5. Bals-Pratsch M, Zietz B, Reichel S, Seifert B. Autoimmune thyreoiditis and fertility services - Consideration for an empirical treatment-concept. Journal fur Reproduktionsmedizin und Endokrinologie. 2005;2:90-5.

6. Beydilli Nacak G, Ozkaya E, Yayla Abide C, Bilgic BE, Devranoglu B, Gokcen Iscan R. The impact of autoimmunity-related early ovarian aging on ICSI cycle outcome. Gynecological Endocrinology. 2018:1-4.

7. Cao M. TPO-AB could increase miscarriage rate of euthyroid ivf women. Fertility and Sterility. 2017;108(3):e212.

8. Cao M, Liu Z, Yang D. The effect of positive TPO-Ab on pregnancy outcomes of euthyroid IVF women. Human Reproduction. 2017;32:i464.

9. Chai J, Yeung WYT, Lee CYV, Li WHR, Ho PC, Ng HYE. The live-birth rates of women with thyroid autoimmunity and/or subclinical hypothyroidism following IVF. Human Reproduction. 2013;28:i322.

10. Chi HB, Qiao J, Wang HN, Hong TP, Gao HW. A clinical study on relationship between thyroid autoimmunity and pregnancy outcomes in in-vitro fertilization women. Human Reproduction. 2012;27.

11. De Brucker M, Unuane D, Velkeniers B, Deridder S, Polyzos N, Bravenboer B, et al. Impact of thyroid autoimmunity on fertility outcome in IVF patients. Human Reproduction. 2016;31:i331.

12. Dokuzeylül Güngör N, Ersahin A, Gorgen B, Cengiz F, Ersahin S, Gungor K. The effects of thyroid autoimmunity presence on ovarian reserve in 117 women with euthyroid state. Human Reproduction. 2015;30:i434-i5.

13. Fumarola A, Grani G, Romanzi D, Del Sordo M, Bianchini M, Aragona A, et al. Thyroid Function in Infertile Patients Undergoing Assisted Reproduction. American Journal of Reproductive Immunology. 2013;70(4):336-41.

14. Geva E, Vardinon N, Lessing JB, Lerner-Geva L, Azem F, Yovel I, et al. Organ-specific autoantibodies are possible markers for reproductive failure: a prospective study in an in-vitro fertilization-embryo transfer programme. Hum Reprod. 1996;11(8):1627-31.

15. Grove-Laugesen D, Ebbehøj E, Knudsen UB. Thyroid status and prevalence of Thyroperoxidase Antibodies (TPO-Ab) in women referred to a danish university fertility clinic. Human Reproduction. 2017;32:i477.

16. Heshiki C, Mekaru K, Miyagi M, Akamine K, Aoki Y. Treatment outcome of In Vitro Fertilization-Embryo transfer in patients who have treated abnormal thyroid function. Journal of Obstetrics and Gynaecology Research. 2018;44(8):1647-8.

17. Karacan M, Alwaeely F, Cebi Z, Berberoglugil M, Batukan M, Ulug M, et al. Effect of antithyroid antibodies on ICSI outcome in antiphospholipid antibody-negative euthyroid women. Reprod Biomed Online. 2013;27(4):376-80.

18. Kim CH, Ahn JW, Kang SP, Kim SH, Chae HD, Kang BM. Effect of levothyroxine treatment on in vitro fertilization and pregnancy outcome in infertile women with subclinical hypothyroidism undergoing in vitro fertilization/intracytoplasmic sperm injection. Fertil Steril. 2011;95(5):1650-4.

19. Kim CH, Chae HD, Kang BM, Chang YS. Influence of antithyroid antibodies in euthyroid women on in vitro fertilization-embryo transfer outcome. Am J Reprod Immunol. 1998;40(1):2-8.

20. Kim MH, Jeong HJ, Kim JH, Cho B, Yun JM, Lee HS. Effect of thyroid autoimmunity in maternal subclinical hypothyroidism. Endocrine Reviews. 2016;37(2).

21. Kotori A, Haxhibeqiri V, Zhuri M, Mulliqi-Kotori V, Haxhibeqiri S. Thyroid disorders in women of reproductive age in Kosovo. Biochimica Clinica. 2013;37:S401.

22. Kutteh WH. Autoimmune factors in assisted reproduction. Minerva Ginecol. 2002;54(3):217-24.

23. Kutteh WH, Schoolcraft WB, Scott RT, Jr. Antithyroid antibodies do not affect pregnancy outcome in women undergoing assisted reproduction. Hum Reprod. 1999;14(11):2886-90.

24. Litwicka K, Arrivi C, Varricchio MT, Mencacci C, Greco E. In women with thyroid autoimmunity, does low-dose prednisolone administration, compared with no adjuvant therapy, improve in vitro fertilization clinical results? J Obstet Gynaecol Res. 2015;41(5):722-8.

25. Medenica S, Garalejic E, Arsic B, Medjo B, Bojovic Jovic D, Abazovic D, et al. Follicular fluid thyroid autoantibodies, thyrotropin, free thyroxine levels and assisted reproductive technology outcome. PLoS One. 2018;13(10):e0206652.

26. Mintziori G, Goulis DG, Gialamas E, Dosopoulos K, Zouzoulas D, Gitas G, et al. Association of TSH concentrations and thyroid autoimmunity with IVF outcome in women with TSH concentrations within normal adult range. Gynecol Obstet Invest. 2014;77(2):84-8.

27. Mintziori G, Goulis DG, Kolibianakis EM, Slavakis A, Bosdou J, Grimbizis G, et al. Thyroid function and autoimmunity during ovarian stimulation for intracytoplasmic sperm injection. Reproduction, fertility, and development. 2017;29(3):603-8.

28. Mintziori G, Kolibianakis EM, Grimbizis GF, Tarlatzis BC, Goulis DG. Thyrart: A Prospective Study on Thyroid Function and Autoimmunity during Ovarian Stimulation for IVF. Endocrine Reviews. 2014;35(3).

29. Monteleone P, Parrini D, Faviana P, Carletti E, Casarosa E, Uccelli A, et al. Female infertility related to thyroid autoimmunity: the ovarian follicle hypothesis. Am J Reprod Immunol. 2011;66(2):108-14.

30. Negro R, Formoso G, Coppola L, Presicce G, Mangieri T, Pezzarossa A, et al. Euthyroid women with autoimmune disease undergoing assisted reproduction technologies: the role of autoimmunity and thyroid function. J Endocrinol Invest. 2007;30(1):3-8.

31. Poppe K, Glinoer D. Thyroid autoimmunity and hypothyroidism before and during pregnancy. Hum Reprod Update. 2003;9(2):149-61.

32. Poppe K, Glinoer D, Tournaye H, Schiettecatte J, Devroey P, van Steirteghem A, et al. Impact of ovarian hyperstimulation on thyroid function in women with and without thyroid autoimmunity. J Clin Endocrinol Metab. 2004;89(8):3808-12.

33. Poppe K, Velkeniers B. Thyroid disorders in infertile women. Ann Endocrinol (Paris). 2003;64(1):45-50.

34. Poppe K, Velkeniers B. Female infertility and the thyroid. Best Pract Res Clin Endocrinol Metab. 2004;18(2):153-65.

35. Reh A, Im S, Amarosa A, Rolnitzky L, Grifo J, Danoff A. Effect of autoimmune thyroid disease (AITD) in older, euthyroid infertile women undergoing in vitro fertilization (IVF). Fertility and Sterility. 2010;94(4):S189-S90.

36. Seungdamrong A, Steiner AZ, Gracia CR, Legro RS, Diamond MP, Coutifaris C, et al. Preconceptional antithyroid peroxidase antibodies, but not thyroid-stimulating hormone, are associated with decreased live birth rates in infertile women. Fertility and Sterility. 2017;108(5):843-50.

37. Seungdamrong AM, Steiner A, Gracia C, Diamond MP, Legro R, Jin S, et al. Antithyroid antibodies, but not thyroid stimulating hormone, are associated with decreased pregnancy rates in infertile women. Fertility and Sterility. 2015;104(3):e36-e7.

38. Singh A, Dantas ZN, Stone SC, Asch RH. Presence of thyroid antibodies in early reproductive failure: biochemical versus clinical pregnancies. Fertil Steril. 1995;63(2):277-81.

39. Tabata C, Fujiwara T, Tsutsumi O. Study on thyroid function and pregnancy rate in assisted reproductive technologies of Japanese women. Endocrine Reviews. 2016;37(2).

40. Unuane D, Velkeniers B, Deridder S, Bravenboer B, Tournaye H, De Brucker M. Impact of thyroid autoimmunity on cumulative delivery rates in in vitro fertilization/intracytoplasmic sperm injection patients. Fertil Steril. 2016;106(1):144-50.

41. Vega M, Barad DH, Seier K, Yu Y, Kushnir VA, Lazzaroni-Tealdi E, et al. Immunologic and inflammatory markers in prediction models of clinical pregnancy and live birth in association with in vitro fertilization (IVF) in women receiving immune-suppressive therapy. Human Reproduction. 2015;30:i305.

42. Wang H, Gao H, Chi H, Zeng L, Xiao W, Wang Y, et al. Effect of Levothyroxine on Miscarriage Among Women With Normal Thyroid Function and Thyroid Autoimmunity Undergoing In Vitro Fertilization and Embryo Transfer: A Randomized Clinical Trial. Jama. 2017;318(22):2190-8.

43. Weghofer A, Himaya E, Kushnir VA, Barad DH, Gleicher N. The impact of thyroid function and thyroid autoimmunity on embryo quality in women with low functional ovarian reserve: a case-control study. Reprod Biol Endocrinol. 2015;13:43.

44. Weghofer A, Himaya E, Kushnir VA, Shohat-Tal A, Barad DH, Gleicher N. Is immune system activation/systemic inflammation a prerequesite for successful reproduction? Association of immune system activation with embryo quality during in vitro fertilization (IVF). Fertility and Sterility. 2013;100(3):S329.

45. Ying Y, Zhong YP, Zhou CQ, Xu YW, Wang Q, Li J, et al. A retrospective study on IVF outcome in patients with anticardiolipin antibody: Effects of methylprednisolone plus low-dose aspirin adjuvant treatment. Journal of Reproductive Immunology. 2012;94(2):196-201.
